# Tony Pawson and the germination and flowering of cell signaling: an appreciation

**DOI:** 10.1186/1741-7007-11-95

**Published:** 2013-08-28

**Authors:** Bruce J Mayer

**Affiliations:** 1 University of Connecticut Health Center, Department of Genetics and Developmental Biology, 400 Farmington Avenue, Farmington, CT 06030, USA

## 

The scientific community was deeply saddened by news of the untimely death of Tony Pawson, a member of the *BMC Biology* editorial board and a leader in the field of cell signaling. I first met Tony when I was a graduate student in the 1980s, at one of those summer research meetings where science was followed by beer and conversation. I still have vivid memories of Tony, with his shock of white hair and ready smile, chatting into the night with students and other colleagues about his current passion, an obscure tyrosine kinase called Fps. Tony exemplified the investigators working on oncogenes at the time - smart, collegial, and burning to understand how cell signaling worked and how it could go so terribly wrong in cancer. In a few short years Tony emerged from this group to become one of the most influential biologists of the past two decades, shaping the new field of cell signaling.

**Figure:**
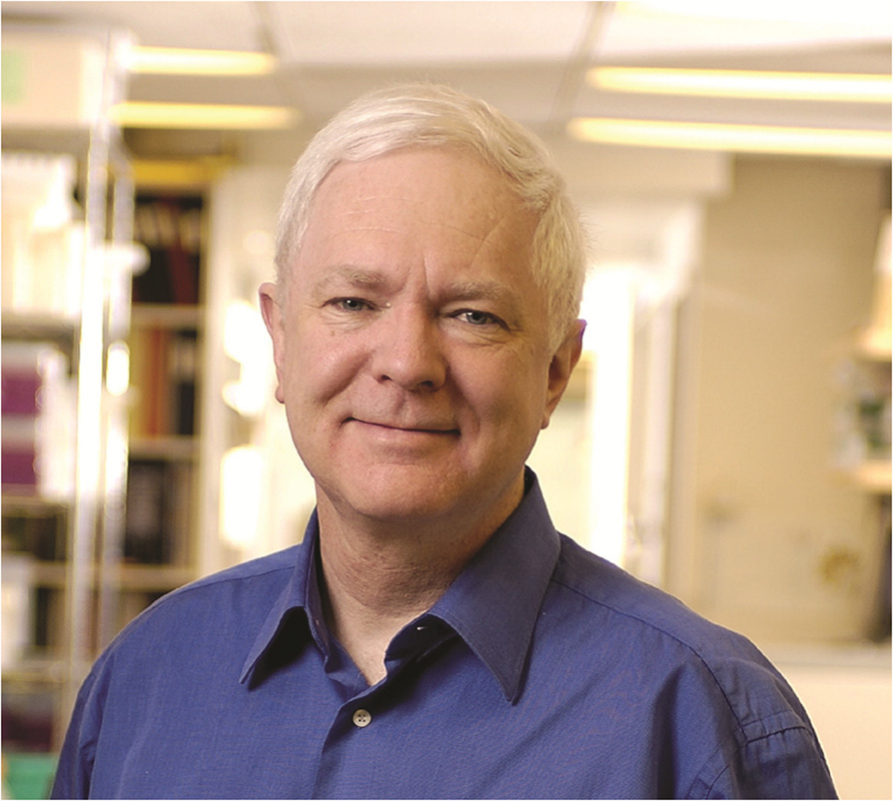
Anthony J Pawson

To understand the significance of Tony’s contributions, we need to think back to how little was known at that time. We knew that unrestrained tyrosine kinase activity could stimulate cells to proliferate, and that the receptors for mitogens such as epidermal growth factor and platelet-derived growth factor also had tyrosine kinase activity. These kinases seemed to work by somehow activating additional signaling proteins such as the G protein Ras and serine/threonine kinases, but we knew almost nothing about the mechanism. How were tyrosine kinases regulated, and how did they transmit downstream signals? How could these mechanisms be subverted in cancer?

Tony’s lab at the time was focused on Fps, which like its much more celebrated cousin, Src, was an oncogenic tyrosine kinase first identified in an avian sarcoma virus. Tony’s group was interested in what caused the activity of the oncogenic kinases to be unregulated compared with that of their unmutated cellular counterparts, and he zeroed in on a region N-terminal to the catalytic domain. In a landmark 1986 paper with Ivan Sadowski (although its importance was hardly appreciated at the time), he coined the term ‘SH2 domain’ for this region, for Src Homology domain 2 - a second region of sequence similarity between Fps and Src, the first being the tyrosine kinase catalytic domain. Soon such ‘homology domains’ began popping up in more and more signaling proteins as they were cloned and sequenced - Ras-GAP, phospholipase C, phosphatidylinositol 3-kinase, and the small oncogenic protein Crk, which seemed to consist of little else. Clearly these small regions had a powerful influence and were widely used in cell signaling, but how might they work?

Taking full advantage of new and improved research tools as they became available (bacterial fusion proteins, X-ray crystallography and NMR spectroscopy, genetic models, high-throughput screening methods), Tony’s group and others quickly demonstrated that SH2 domains bound to specific tyrosine-phosphorylated peptides and thus served to relocalize cytosolic effector proteins containing SH2 domains to the membrane upon activation of membrane-associated tyrosine kinases. Other modular signaling domains were soon found to mediate specific interactions with other peptides or lipids. These discoveries were quite stunning at the time, hinting at an entirely new and unexpected paradigm for signaling. But beyond the mere details of mechanism, Tony was the one who saw the bigger picture most clearly - the fundamental importance of protein-protein interactions and subcellular localization as essential currencies of information transfer. He also saw how modular protein and lipid interaction domains provided a credible means for signal transduction pathways to emerge and gain new functions in the course of evolution. More than anyone else, Tony made sense of the new discoveries and revealed the underlying logic for all to see.

It is hard to overstate the excitement of those times. The answers to big, important questions came tumbling out one after the other, as disparate pieces of evidence from the fields of biochemistry, genetics, virology, and developmental biology began to fit together in a profoundly satisfying way. And through it all Tony led the way - through the work of his lab, through his beautifully written and deeply insightful review articles, and through his willingness to share what he knew with his colleagues.

In the decades that followed these initial discoveries, most of those involved moved on, or focused on ever-smaller pieces of the puzzle. By contrast, Tony took on the more challenging task of building on those results on a grand scale. In his position as director of the Lunenfeld Research Institute in Toronto, he worked to assemble large interdisciplinary teams that were needed to tackle the questions he wanted to address. He was a pioneer in incorporating emerging disciplines and technologies such as bioinformatics and mass spectrometry into signaling research. In short, he made Toronto the center of the signaling universe. He had a big vision and the skills, energy, and intellect to pursue that vision, probably better than anyone else in the field.

While Tony’s scientific accomplishments were immense, they cannot explain why so many of us will miss him so keenly. Above all, Tony was a wonderful colleague and friend. I can personally attest that he was always far kinder to me than I had any reason to expect, given my position as a junior investigator directly competing with his lab. He provided wise advice, shared his lab’s results before publication, collaborated when possible, and gladly acknowledged and appreciated the work of others. He was a genuinely selfless and humble man, terms that are not often associated with scientists of Tony’s stature. Although he was aware of his own accomplishments, he never let it go to his head (he loved to point out that he had been awarded the Noble Prize - a relatively obscure Canadian research award - knowing full well that he was perennially touted as a shoo-in for the more celebrated Swedish prize).

I got to know Tony better in recent years as we worked together on a textbook of cell signaling. The clarity of his vision and his comprehensive knowledge of the field were the solid foundation upon which the book is built. Sadly, personal tragedies, particularly the wrenching loss of his beloved wife Maggie to cancer, made it more difficult for Tony to focus on science in the past few years. It is a reminder that despite our talents, accomplishments, and essential goodness, we all live close to the edge of disaster. But although we lost Tony far too soon, we can still take joy in all that he accomplished while he was with us: the many trainees, colleagues and friends whose lives he touched, the new insights and discoveries he brought to light, the institutions and ideas that he fostered. Many of us will feel that the world is a far better place because of Tony Pawson.

